# Post-malaria neurological syndrome (PMNS): a rare case report with brain biopsy findings

**DOI:** 10.1186/s12879-023-08704-z

**Published:** 2023-12-19

**Authors:** Mouhammad J Alawad, Moustafa Almayoof, Adel Al bozom, Taha Alkhidir, Saeed S Emam, Khalifa Farfar

**Affiliations:** 1 Department of Medical Education, Internal Medicine Residency Program, Hamad Medical Corporation, Doha, 3050 Qatar; 2 Department of Medicine, Division of Neurology, Hamad Medical Corporation, Doha, Qatar; 3 Department of clinical radiology, Hamad Medical Corporation, Doha, Qatar; 4 Department of Medicine, Al Wakra hospital, Hamad Medical Corporation, Doha, Qatar

**Keywords:** Post Malaria neurological syndrome (PMNS), Malaria, Brain biopsy, Acute disseminated encephalomyelitis (ADEM)

## Abstract

Post-malaria neurological syndrome (PMNS) is a rare, self-limiting condition that presents with a wide range of neurological manifestations after clearance of malarial infection, especially 𝘗𝘭𝘢𝘴𝘮𝘰𝘥𝘪𝘶𝘮 f𝘢𝘭𝘤𝘪𝘱𝘢𝘳𝘶𝘮, most patients recover without residual deficits. Here we present a case of a 29-year-old, male with a recent history of malaria treated successfully, who presented due to a generalized tonic-clonic seizure, without any other neurological symptoms, the examination and labs were unremarkable, he underwent a computer tomography (CT) scan and Magnetic resonant imaging (MRI) which both showed two areas of vasogenic edema involving the subcortical white matter of left frontal and right posterior parasagittal regions, all autoimmune screens, infection workup from blood and CSF were negative, he underwent a brain biopsy that showed intense perivascular inflammation with neuronal loss and gliosis, findings are nonspecific and can be seen in a variety of condition. The patient’s condition improved, and he was discharged without any complications.

## Introduction

Malaria is an endemic disease with high morbidity and mortality if not treated. It can present with de novo neurological symptoms and can lead to sequelae, especially with cerebral malaria. Post-malaria neurological syndrome (PMNS), on the other hand, is the development of new neurological manifestations in patients who had a recent malaria infection with successful treatment, PMNS is a relatively new phenomenon [1], with low incidence, the presentation has a wide range of neurological signs and symptoms from headache to seizure, confusion, and loss of consciousness, and a set of criteria was suggested to aid in the diagnosis of PMNS, requiring a recent malaria infection that was successfully treated by malaria negative smear and a symptom-free period after malaria cure [2]. Before considering PMNS, all efforts must be made to rule out other causes that can explain the patient presentation; the presence of abnormal brain imaging findings was reported in up to half of the cases with PMNS with no specific pattern or distribution [3]; almost all cases showed neurological improvement and resolution of all symptoms without remaining deficits, although steroids were used in some severe cases to accelerate recovery. However, its use is still debated as to whether it plays a role in the improvement of these cases [1, 4]. Herein, we present a case of new seizure disorder in a patient with a recent Malaria infection, The importance of our case is to highlight the PMNS brain biopsy findings, which have not been reported before, this can lead to a better understanding of the disease. We report this case to highlight PMNS as one of the differential diagnoses for any patient presenting with neurological manifestations after a cured malaria infection.

## Case presentation

A 29-year-old black African immunocompetent male from Ghana with no history of chronic diseases recently came to Qatar, living in Ghana before coming to Qatar, and did not leave his home country. Two months earlier, he was diagnosed with malaria and treated successfully in his home country, little information was available about his illness in Ghana, and no scripts were available. The patient presented to the hospital because of a generalized tonic-clonic seizure that occurred for the first time at home and was witnessed by his friend, followed by postictal confusion that resolved when the patient arrived at the hospital; the patient complained of a mild headache; otherwise, he denied weakness, numbness, history of seizure, blurred vision, hearing loss, or loss of consciousness.

**Fig. 1:**
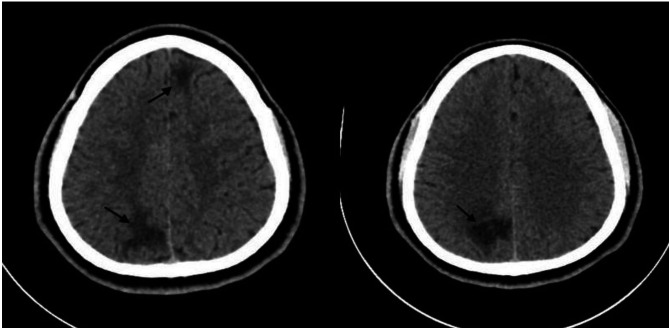
CT head showed two areas of hypodensity (black arrows) lesions representing vasogenic oedema involving the subcortical white matter of the left frontal and right posterior parasagittal location

**Fig. 2:**
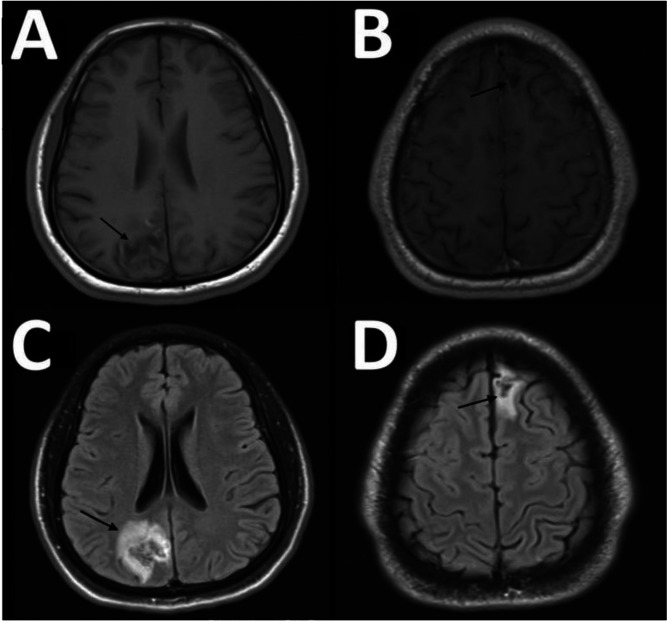
MRI head T1 (**A**, **B**), Flair (**C**, **D**) showed two lesions (black arrows) with heterogenous area seen mainly in subcortical and abutting cortical noted on right occipital parietal and anterior paraflacine left frontal lobe with surrounding marked vasogenic edema

Vitally was stable, and labs including CBC, electrolyte, Renal, and liver function were within normal, two malaria blood test films came negative, and the patient had high creatinine kinase, and myoglobulin on admission related to seizure which improved after, a computed tomography (CT) head (Fig. 1) showed two areas of vasogenic edema involving the subcortical white matter of the left frontal and right posterior parasagittal location with subtle increased adjacent leptomeningeal enhancement, a magnetic resonance imaging (MRI) head with contrast (Fig. 2A, B, C, and D) showed heterogenous area seen mainly in subcortical and abutting cortical noted on right occipital parietal and anterior para-flacine left frontal lobe with surrounding marked vasogenic edema. The CSF opening pressure was 19 mm for H2o and analysis showed WBC:2, Glucose:3.4 mmol/l, protein:0.41 gm/l, no oligoclonal bands, culture: negative, TB smear, culture: negative, viral PCR: (HSV1,2, varicella zoster, enterovirus, and mumps) were negative, negative for oligoclonal bands, and cytology negative for malignancy; CSF IgG index 0.5 (within normal range); tests for brucella, syphilis, Schistosoma, HIV Ag/Ab were negative, autoimmune screening including ANA, ANCA, RF, C3, and C4 were all negative; QuantiFERON, blood culture also negative; the patient was started on phenytoin, no corticosteroids were administered, and was doing well throughout the hospital course without any complaints.

**Fig. 3:**
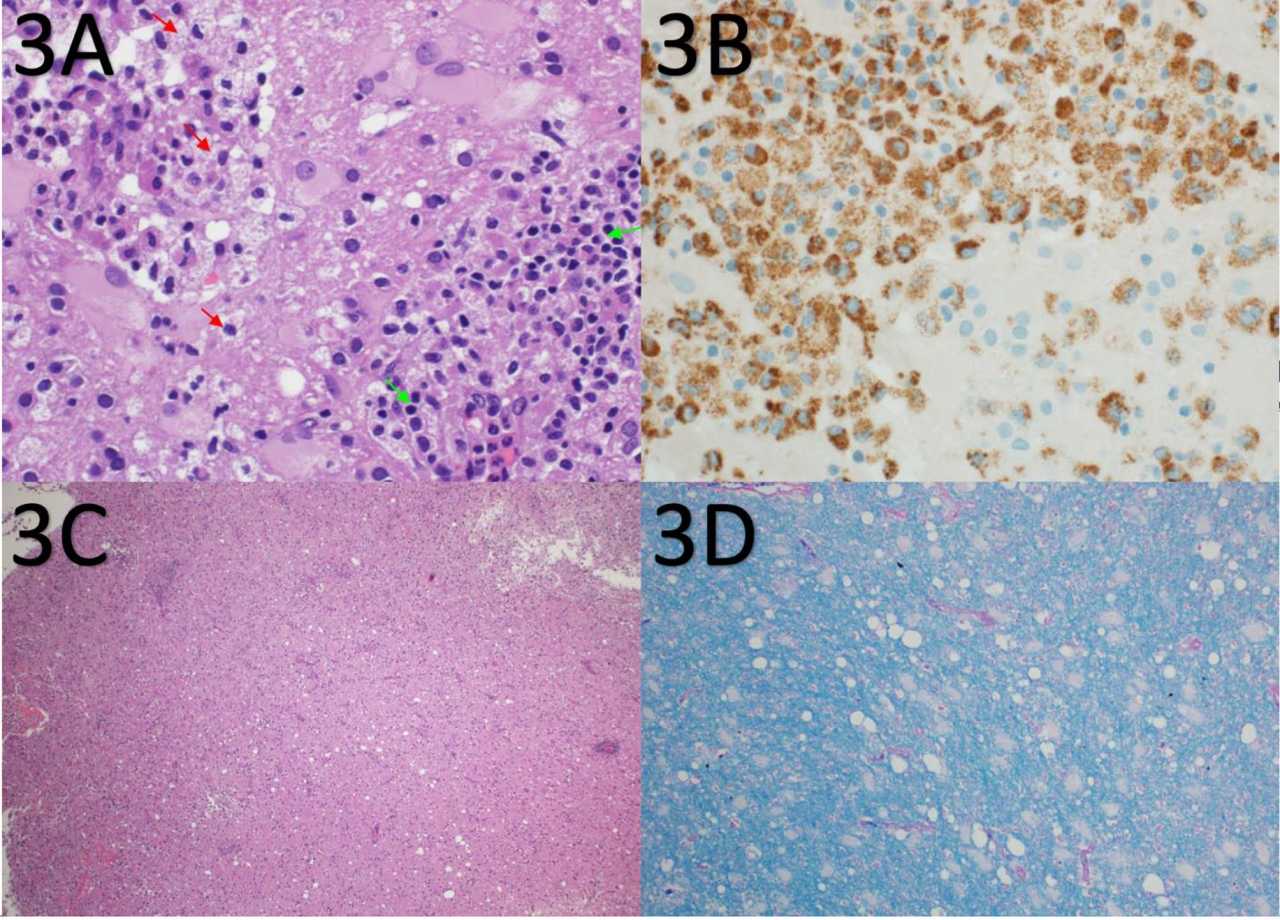
**A**) Microscopic examination reveals brain tissue heavily infiltrated by macrophages (red arrows) and lymphocytes (green arrows) (H&E ×400). **B**): Immunohistochemistry using antibodies against CD68 highlights the numerous macrophages infiltrating brain tissue (immunohistochemistry against CD68 × 400). **C**): Low power view showing the destructive and cavitary inflammatory brain lesion (H&E ×40). **D**): Special stain for myelin (Luxol Fast Blue) highlights the presence of intact myelin within the brain parenchyma (Luxol Fast Blue ×200)

**Fig. 4:**
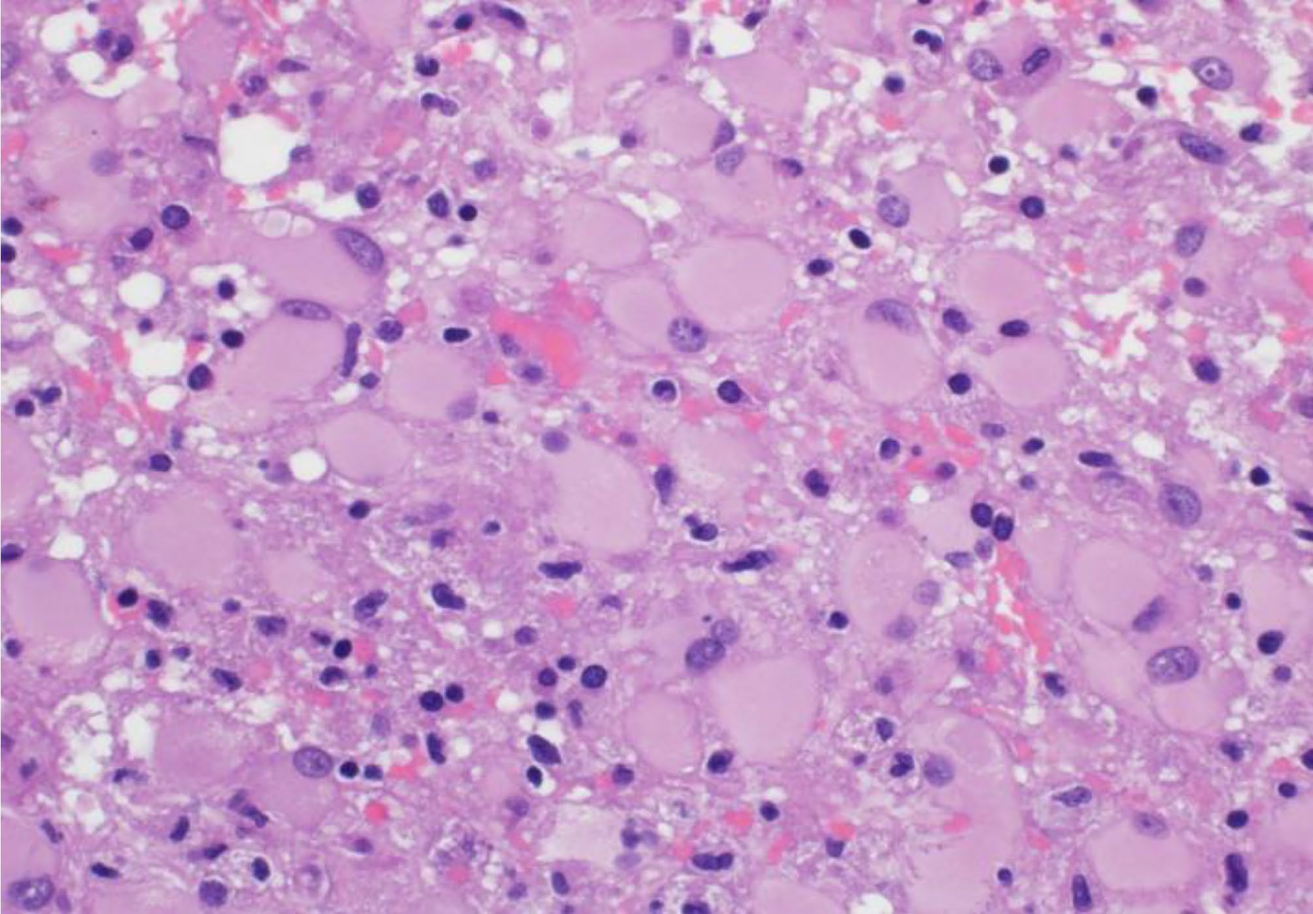
Numerous gemistocytes compatible with reactive astrocytosis (H&E ×400)

**Fig. 5:**
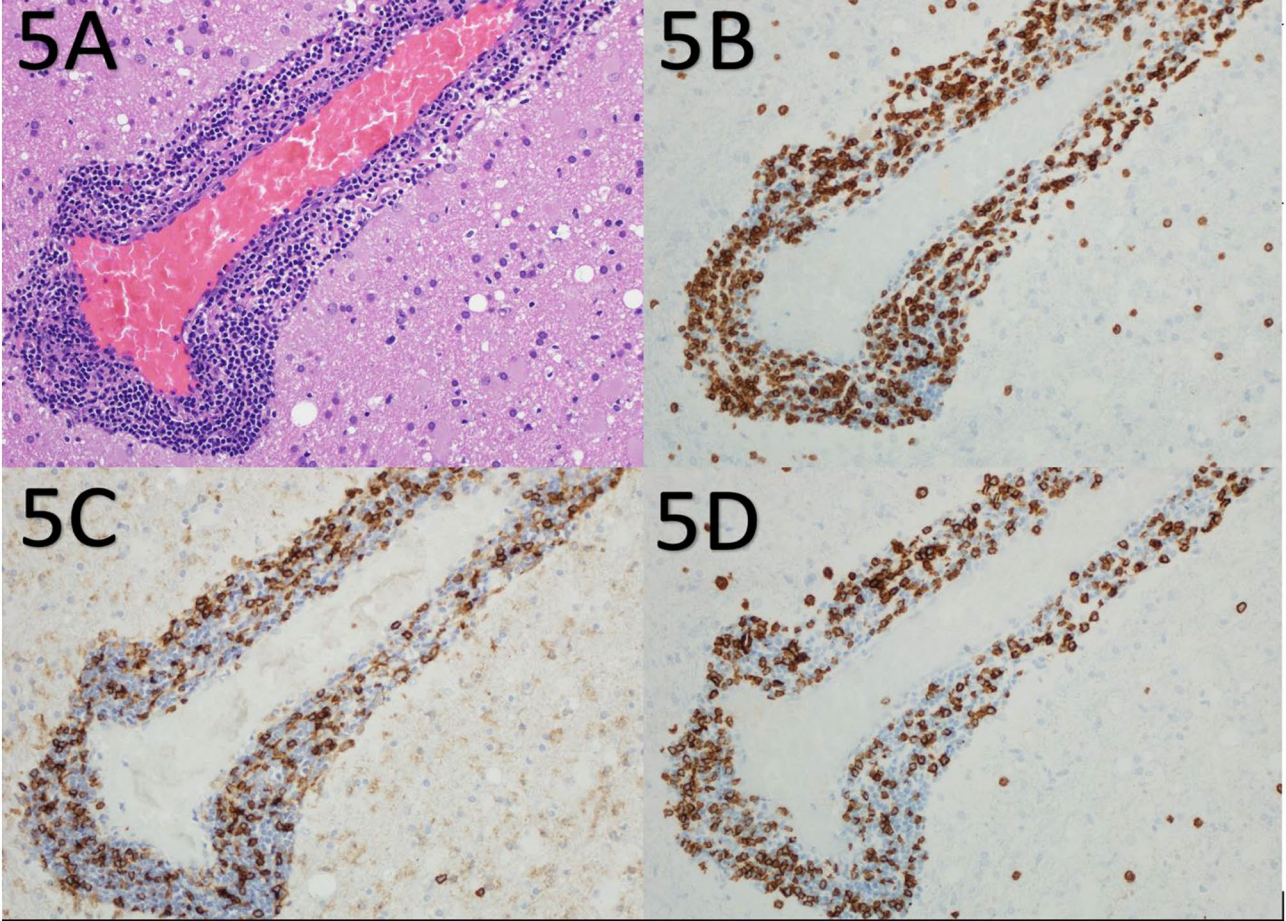
**A**): Perivascular cuffing by lymphocytes (H&E ×400). **B**): Perivascular cuffing by T lymphocytes (immunohistochemistry against CD3, ×400). **C**): Perivascular cuffing by T lymphocytes (immunohistochemistry against CD4, ×400). **D**): Perivascular cuffing by T lymphocytes (immunohistochemistry against CD8, ×400)

Given the patient’s age and imaging findings, serious conditions such as primary malignancy, metastasis, or hidden infection need to be excluded, and brain biopsy was performed as advised by the neurology team from the frontal lesion. Histological examination demonstrated extensive and diffuse lymphohistiocytic inflammation in the cerebral cortex and white matter, areas of parenchymal injury, and destruction. The inflammatory infiltrate was composed predominantly of macrophages and activated microglia as well as small lymphocytes, predominantly small T cells highlighted by CD3 (Figs. 3A and B and 4B) with a reversed CD4/CD8 ratio (Fig. 4 C and 4D). Only a few small B cells were identified. Areas of parenchymal destruction showed cavitation and cystic changes (Fig. 3C) with extensive reactive gliosis characterized by astrocytes with eccentric nuclei and abundant eosinophilic cytoplasm (gemistocytes) (Fig. 5). The vessels showed prominent perivascular inflammatory infiltrates with a greater number of venules than the arteries and arterioles (Fig. 4A and B). The vessels did not exhibit intimal hyperplasia, thinning of the media, fibrinoid necrosis, luminal narrowing, or luminal thrombosis. The vessels had intact elastic layers. Luxol Fast Blue staining for myelin did not show myelin loss (Fig. 3D). There was no definite evidence of vascular necrosis, granulomas, acute inflammation, emperipolesis, viral inclusions, parasites, Plasmodium, fungal elements, demyelination, or malignancy. Immunohistochemical staining was negative for BRAF V600E, HSV-1, HSV-2, and CMV.

The patient was discharged home after the brain biopsy and had a follow-up MRI one month after the biopsy, which showed a postoperative surgical cavity measuring 27 mm in diameter. It shows extensive blooming on SWI, denoting a hemorrhagic component, and the left frontal lesion is likely totally removed; otherwise, the patient is stable without any complaint. He continued to attend regular follow-ups. After discharge, the patient attended a follow-up at the neurology clinic at one month then 3 months interval with no apparent neurological deficit or reported any symptoms.

## Discussion

Post-malaria neurological syndrome (PMNS) is a relatively rare transient clinical entity, post malaria neurological symptoms were first reported in early 1990 [1]; however, the definition of *the post-malaria neurological syndrome* was first presented by Nguyen et al. in their study as a collection of new neurological or psychiatric features that occur after full recovery of severe Falciparum Malaria infection [2] Since then, cases of PMNS have been reported sporadically, mostly after Malaria Falciparum but also after infection with Vivax species [3], which is more notable after severe Malaria infection [5]; the time frame requires a symptom-free period varying from days after recovery to months [6, 7], and the latency period can extend to more than 100 days post-infection [8]. This is followed by the complete resolution of symptoms without sequelae [5].

The relative risk for the development of PMNS ranges between 0.7 and 1.8 per 1000 cases [2, 9], while malaria is a prevalent disease in endemic areas (500 million annually), suggesting that PMNS is underreported as only severe or serious manifestations are encountered [10].

The symptoms of PMNS encompass a wide spectrum of neurological or psychiatric symptoms, most commonly with acute psychosis like confusion 66–72% [1, 10] and seizures (~ 30%) [11]. To correctly identify PMNS, a negative blood smear showing clearance of parasitemia is necessary for diagnosis [2, 12]. A workup should be undertaken to rule out any other possible explanations, such as viral, bacterial, autoimmune, or malignancy. CSF analysis of the cerebral spinal fluid showed pleocytosis with lymphocyte predominance and increased protein levels [7, 8]. However, no relationship was found between symptom severity and worsening CSF findings [2].

The exact mechanism of PMNS remains elusive, and few theories have tried to explain it. One of the first suggested etiologies is that of molecular mimicry, in which antibodies directed towards the malaria antigens would cross-react with autoantigens [3, 5]. However, this would require a prolonged persistent infection or relapse, which by default would not fit under the definition of PMNS [8]. Another theory about structural aberrations caused by the recent malaria infection could explain the pathogenesis. However, the hallmark of rapid and complete resolution of symptoms and the time frame makes it unlikely [3, 11]. Other possibilities include co-infection with a viral agent that precipitates encephalitis or neurotoxicity as an adverse drug reaction [5].

Interestingly, all the reported cases seem to adopt an immunological process as the main pathogenesis mechanism [6, 11] supported by the symptom-free period and markers of immune system activity during the disease; Poulet described multiple possible antibodies implicated in the development of PMNS-like *voltage-gated potassium channels* (VGKC). The proposed management lines would further support this theory [3, 8].

Imaging modalities such as magnetic resonance imaging (MRI) have been reported to be abnormal in less than 50% of cases [7]. The findings showed nonspecific changes with increased uptake in the gray and white matter [5]. However, it is not clear how long the changes persist when MRI findings are abnormal. To the best of our knowledge, no previous case of PMNS underwent a brain biopsy. Although the biopsy characteristics revealed a nonspecific neuronal injury and inflammatory response, it was crucial to eliminate the possibility of an underlying malignancy or an autoimmune process such as vasculitis or ongoing infection. No final conclusion can be drawn from the biopsy findings, but it seems that the dense infiltration with lymphocytes and macrophages, most likely in settings of immunological activation, and their interaction with the brain parenchyma are the leading causes of the pathogenesis of the symptoms.

Based on available literature it seems that there is difference between the pathological findings of cerebral malaria and that of post malaria neurological syndrome, our case is the only one to provide pathological aspects of PNMS, however, in cerebral malaria, one hallmark would be the sequestered RBC, which can appear adherent to the endothelium in some cases, the endothelial cells also appeared hypertrophied with fibrin necrosis & microvascular thrombosis, another difference is myelin loss [13] which was not observed in our case, cerebral malaria may have features of blood brain barrier leakage [13] but in our case there is no features of vascular leakage, the perivascular infiltrate in our case demonstrated a predominance of lymphocytes, macrophages & activated microglia which is different from what has been observed in cerebral malaria where monocyte & macrophages are predominant with scanty CD8 in the vascular lumen [13], the pathological pattern suggest a parenchymal injury & reactive gliosis (Gemistocytes) so it seems that the injury goes beyond the blood brain barrier.

PMNS has common features with Acute disseminated encephalomyelitis (ADEM) and autoimmune encephalitis (AIE); the symptomology, time frame, lab, imaging findings, and prognosis would place PMNS between ADEM and AIP [1] with possible overlapping traits from both syndromes. In ADEM, there are features of inflammation and myelin loss, with proliferation of endothelial cells and fibrinoid necrosis [14], which is not apparent in our case, and the perivascular infiltrate could be similar in post malaria neurological syndrome and ADEM, as both have CD + 4 and CD + 7. PMNS is self-limiting and spontaneous full recovery is a rule. However, the use of steroids in prolonged or severe cases was proposed as the first-line treatment, with immunoglobulins (IVIG) and plasmapheresis coming next in line [8, 11].

## Conclusion

Post-malaria neurological syndrome is an important complication of Malaria infection, characterized by a post-infection symptom-free period followed by de novo neuropsychiatric manifestations that would subside completely within a few days to weeks without residual symptoms. However, the syndrome is quite rare and may be underreported, and further reports are needed to shed light on the possible etiologies, the natural course of the disease, interventions, and long-term prognosis.

## Data Availability

Data and materials are available on request.
